# Lymphoproliferative disorder during temozolomide therapy; a representative case of a formidable complication and management challenges

**DOI:** 10.1186/s12883-023-03274-8

**Published:** 2023-06-09

**Authors:** Daisuke Sato, Hirokazu Takami, Shunsaku Takayanagi, Kazuki Taoka, Mariko Tanaka, Reiko Matsuura, Shota Tanaka, Nobuhito Saito

**Affiliations:** 1 Department of Neurosurgery, The University of Tokyo Hospital, 7-3-1 Hongo, Bunkyo-Ku, Tokyo, 113-8655 Japan; 2 Department of Hematology and Oncology, The University of Tokyo Hospital, Tokyo, Japan; 3 Department of Pathology, The University of Tokyo Hospital, Tokyo, Japan

**Keywords:** Lymphoproliferative disorder, Glioma, Temozolomide, Epstein-Barr virus

## Abstract

**Background:**

Lymphoproliferative disorder represents a heterogeneous clinicopathological spectrum characterized by uncontrolled proliferation of lymphocytes. Immunodeficiency is a major trigger of its development. While induction of immunodeficiency is a well-known adverse effect of temozolomide therapy, development of lymphoproliferative disorder following temozolomide therapy has not previously been described.

**Case presentation:**

A patient with brainstem glioma developed constitutional symptoms, pancytopenia, splenomegaly and generalized lymphadenopathy during the 2nd cycle of maintenance therapy following induction therapy with temozolomide. Epstein-Barr virus-infected lymphocytes were observed histopathologically and “other iatrogenic immunodeficiency-associated lymphoproliferative disorder” (OIIA-LPD) was diagnosed. Although discontinuation of temozolomide led to rapid remission, relapse was observed 4 months later. CHOP chemotherapy was induced, resulting in secondary remission. Vigilant follow-up for another 14 months showed radiologically stable brainstem glioma and no further recurrence of OIIA-LPD.

**Conclusions:**

This is the first report documenting OIIA-LPD during temozolomide administration. Timely diagnosis of the disease and discontinuation of the causative agent were considered to be the management of choice. Close monitoring for relapse should be continued. Finding a balance between glioma management and controlling the remission of OIIA-LPD remains to be clarified.

**Supplementary Information:**

The online version contains supplementary material available at 10.1186/s12883-023-03274-8.

## Background

The term “lymphoproliferative disorder” (LPD) covers a heterogeneous clinicopathological spectrum characterized by uncontrolled proliferation of lymphocytes [1]. This entity ranges from non-neoplastic lymphoid proliferation to malignant lymphoma that can be discerned by the clonality of the proliferating lymphocytes [2]. The World Health Organization (WHO) classifies LPD into four categories: post-transplant lymphoproliferative disorders; lymphomas associated with HIV (human immunodeficiency virus) infection; lymphoproliferative disorders associated with primary immune disorders; and other iatrogenic immunodeficiency-associated lymphoproliferative disorders (OIIA-LPD) [2]. OIIA-LPD is a category that represents LPD arising in patients treated with immunosuppressants such as methotrexate, or in patients with immunosuppressed conditions other than the post-transplant setting [3, 4]. Causative relationships of Epstein-Barr virus (EBV) activation to OIIA-LPD have repeatedly been described [3, 5, 6].

Temozolomide has been the mainstay of treatment for diffuse glioma since the notable study by Stupp et al. in 2005 [7]. Temozolomide is an alkylating agent with high bioavailability and considerable blood–brain barrier penetration, well-known for its excellent efficacy and little adverse effects [8]. Constipation and epigastric discomfort are common side effects, and while hematologic toxicity is a recognized adverse effect, its frequency is low [9]. Temozolomide-induced aplastic anemia (TIAA) is one of the hematologic toxicities that has recently been garnering attention and has gained notoriety for its profound cytopenia [8]. The leukemogenic potential of temozolomide has also been noted, and development of acute myeloid leukemia, acute lymphoblastic leukemia or myelodysplastic syndrome has been reported as late as 5–10 years after exposure [10–13]. However, there is a paucity of evidence regarding the occurrence of LPD as a side effect of temozolomide therapy.

We encountered a case of diffuse astrocytoma in the brainstem treated with induction and maintenance temozolomide, leading to profound cytopenia, followed by the development of OIIA-LPD. This appears to represent the first report of OIIA-LPD subsequent to temozolomide administration. We illustrate the presentations before and after its development, disease management, and our considerations with regard to finding the balance between glioma treatment and control of the hematologic disorder.

## Case presentation

**Fig. 1:**
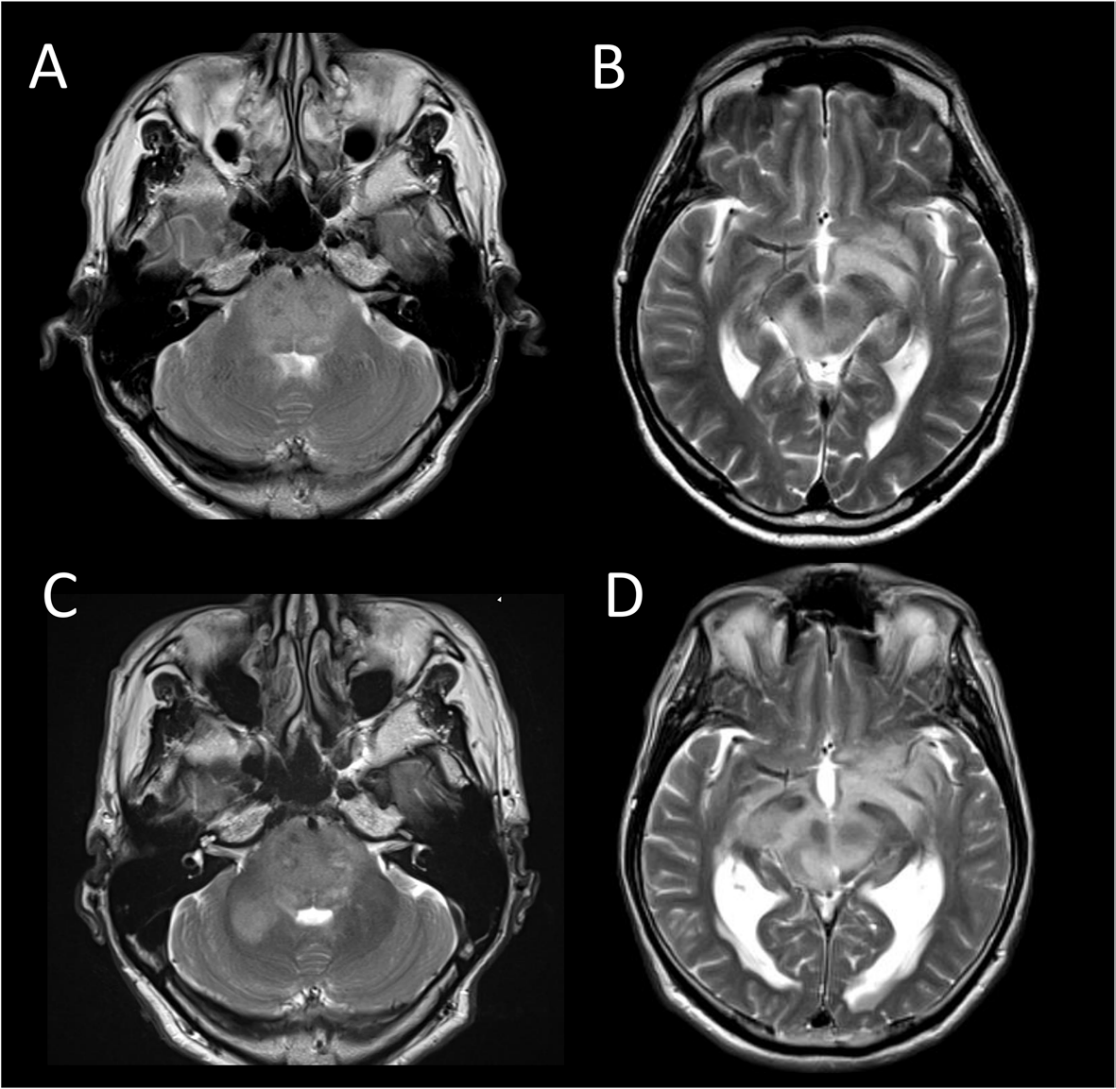
The patient presented with high-intensity lesions on T2-weighted imaging of the pons (**A**). The lesion extended to the midbrain (**B**). The patient initially preferred to be followed-up at an outpatient clinic, during which time the lesion extended to the right middle cerebellar peduncle (**C**) and involved the midbrain and thalamus bilaterally (**D**)

A 70-year-old man with no contributory medical history presented to the outpatient clinic complaining of a 4-year history of dysgeusia. Magnetic resonance imaging (MRI) revealed a space-occupying lesion appearing hyperintense on T2-weighted imaging in the pons (Fig. 1A). The area of abnormal signal intensity extended to the midbrain (Fig. 1B). The patient initially preferred to be followed-up with imaging, during which time mild impairment of deglutition and memory disturbance gradually developed. During the course of 2 years, the lesion extended from the pons to the right middle cerebellar peduncle (Fig. 1C) and invaded the midbrain and thalamus (Fig. 1D), followed by the development of obstructive hydrocephalus.

**Fig. 2:**
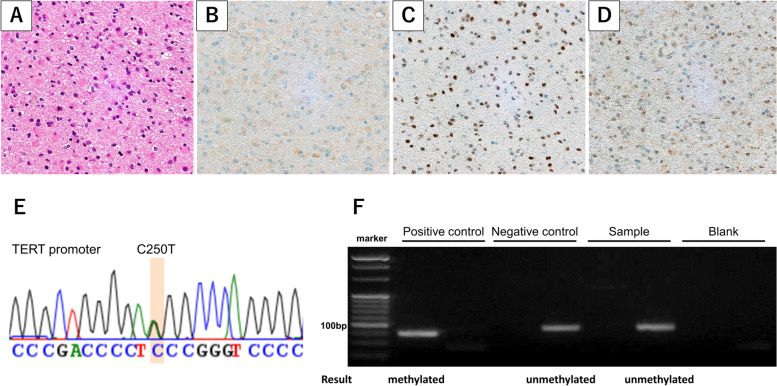
Stereotactic biopsy was performed, revealing diffuse infiltration of pleomorphic cells with nuclear atypia and eosinophilic cytoplasm (**A**). Immunohistochemical staining for R132H IDH1 yielded negative results (**B**). ATRX positivity was retained (**C**). Negative results were obtained for H3K27M (**D**). Sanger sequencing of the TERT promoter revealed C250T mutation (**E**). Methylation-specific polymerase chain reaction demonstrated unmethylated MGMT promoter. Results for the positive control, negative control, sample and blank are displayed from left to right (**F**). The samples derive from the same experiment and the gels and blots were processed in parallel. The diagnosis was glioblastoma, IDH-wildtype

As diffuse glioma was strongly suspected, he underwent endoscopic third ventriculostomy together with stereotactic biopsy of the brainstem lesion. Histopathological investigations showed proliferation of pleomorphic glial cells with nuclear atypia (Fig. 2A). Negative results for IDH R132H were obtained with immunohistochemical staining (Fig. 2B). ATRX positivity was retained (Fig. 2C). Tumor cells were negative for H3K27M (Fig. 2D).

Tumor DNA was extracted from frozen tumor tissue using a DNeasy Blood & Tissue Kit (Qiagen, Tokyo, Japan). *IDH1* R132 and *IDH2* R172 were analyzed by Sanger sequencing, as previously reported [14, 15], revealing no hotspot mutations. The two mutation hotspots in the *TERT* promoter were analyzed in tumor DNA by Sanger sequencing, as previously reported [16], showing the presence of C250T mutation (Fig. 2E). Methylation-specific polymerase chain reaction was performed in the following steps. A bisulfite modification was performed using 400 ng of genomic DNA from frozen tissues using an EZ DNA methylation-Gold Kit (Zymo Research Corporation, Irvine, CA) according to the protocol provided by the manufacturer. Primer sequences were: 5'-TTTCGACGTTCGTAGGTTTTCGC-3' (forward) and 5'-GCACTCTTCCGAAAACGAAACG-3' (reverse) for methylated product; and 5'-TTGTGTTTTGATGTTTGTAGGTTTTTGT-3' (forward) and 5'-AACTCCACACTCTTCCAAAAACAAAACA-3' (reverse) for unmethylated product. Polymerase chain reaction (PCR) was performed using TAKARA EpiTaq (Takara Bio, Tokyo, Japan) according to the protocol from the manufacturer. Initial denaturation was performed at 94 °C for 30 s, followed by 37 cycles of amplification consisting of denaturation at 94 °C for 20 s, annealing at 59 °C for 30 s and extension at 72 °C for 30 s, with a final elongation at 72 °C for 2 min. TAKARA 3522 EpiScope Methylated HCT116 Genomic DNA (Takara Bio) and TAKARA 3521 EpiScope Unmethylated HCT116DKO Genomic DNA (Takara Bio) were used as positive and negative controls, respectively. The results demonstrated that the present case harbored an unmethylated *MGMT* promoter (Fig. 2F). Taking all these findings together, the patient was diagnosed with glioblastoma, IDH-wildtype, CNS WHO grade 4.

Chemoradiotherapy was implemented based on the Stupp regimen, as radiation (54 Gy in 27 fractions) with concomitant temozolomide (75 mg/m^2^/day) for a 30-day period. After completing this regimen, the patient was continued on maintenance temozolomide at the outpatient clinic.

**Fig. 3:**
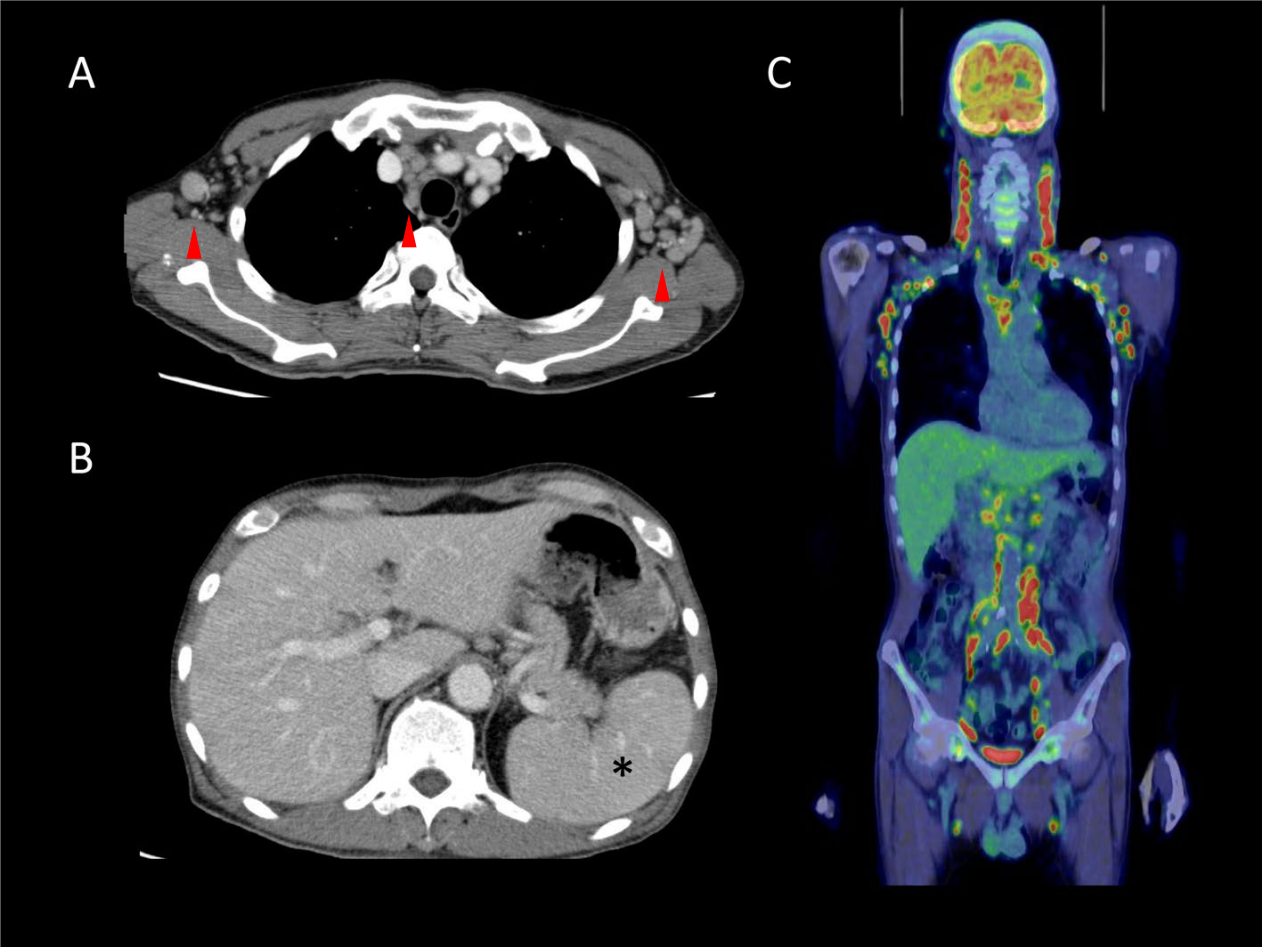
During the 2nd cycle of maintenance therapy following induction therapy with temozolomide, the patient experienced constitutional symptoms and cytopenia. Computed tomography showed systemic lymphadenopathy (**A**; arrowhead) and splenomegaly (**B**; asterisk). Positron emission tomography showed marked fluorodeoxyglucose-avid lymph nodes in the neck, axillae, mediastinum and para-aortic region (**C**)

Five months after histopathological diagnosis and following 2 cycles of temozolomide maintenance treatment, the patient reported constitutional symptoms including fever, anorexia and general malaise. Physical examination showed systemic lymphadenopathy (Fig. 3A, arrowhead) and splenomegaly (Fig. 3B, asterisk). Positron emission tomography-computed tomography exhibited marked avidity for fluorodeoxyglucose in lymph nodes of the neck, axillae, mediastinum and para-aorta region (Fig. 3C).

**Fig. 4:**
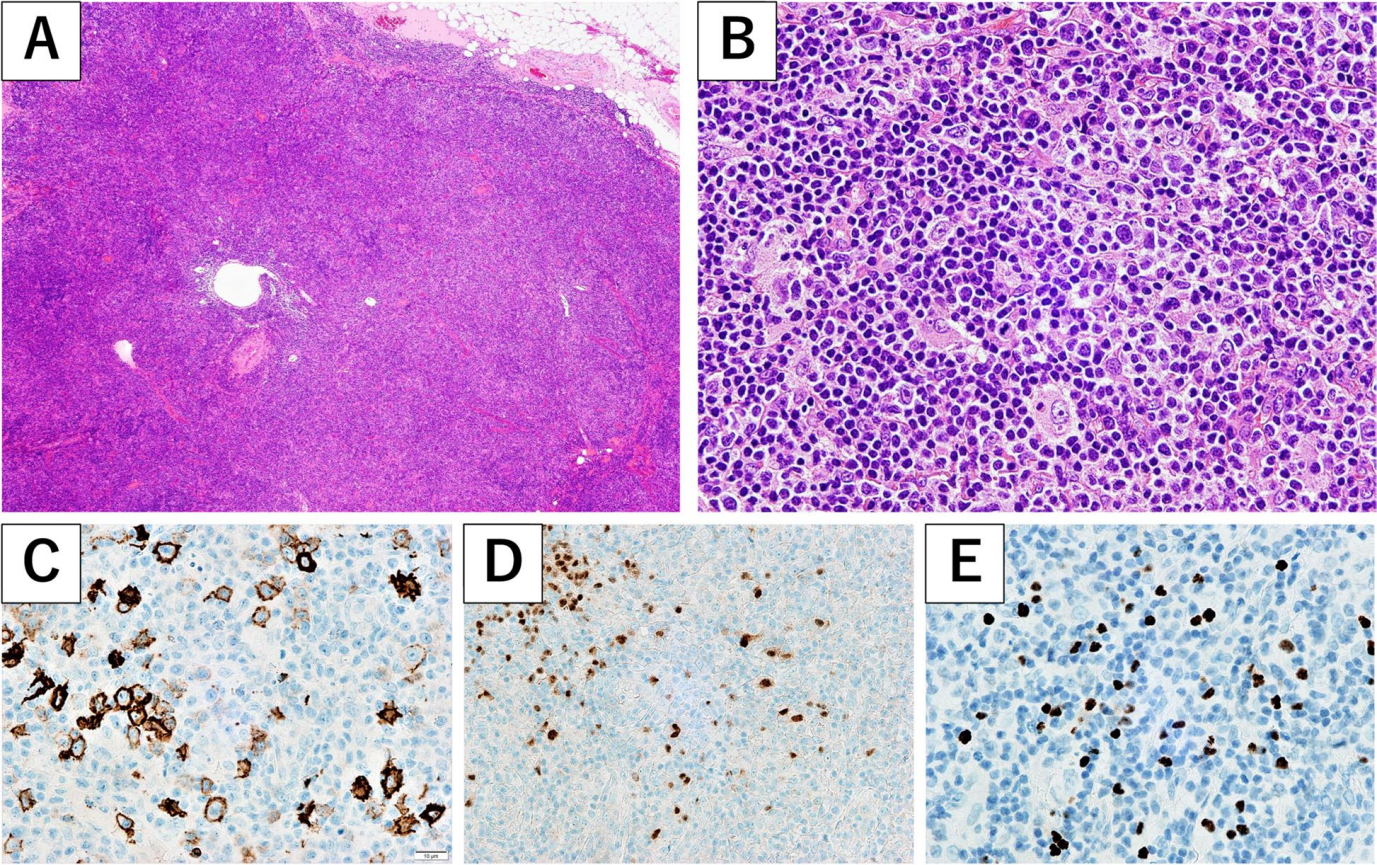
Histopathological investigation of an enlarged cervical lymph node revealed dense infiltration of lymphocytes, and the normal lymph node architecture was widely effaced (**A**). Infiltration of small to medium-sized lymphocytes, plasma cells and histiocytes was observed (**B**). Large, atypical lymphocytes with prominent nucleoli were sparsely observed. These large lymphocytes were positive for CD20 (**C**) and PAX5 (**D**). Epstein-Barr virus (EBV) DNA was confirmed by *in-situ* hybridization (**E**)

Blood examinations revealed profound cytopenia and hyponatremia. Platelet count was decreased to 20 × 10^3^/µL on admission. Although hemoglobin and white blood cell count were both within normal limits at first, hemoglobin decreased from 12.5 g/dL to 9.3 g/dL and white blood cell count decreased from 7400/µL to 2900/µL within one week. Atypical lymphoid cells resembling plasmacytes were also observed in peripheral blood smears, and soluble interleukin-2 receptor (IL-2R) was elevated to over 9000 IU/L. An enlarged cervical lymph node was biopsied for diagnostic evaluation. Pathologically, the lymph node architecture was widely effaced (Fig. 4A) with infiltration of small to medium-sized lymphocytes, plasma cells and histiocytes (Fig. 4B). Large, atypical lymphocytes with prominent nucleoli were sparsely observed. These large lymphocytes were positive for CD20 (Fig. 4C), PAX5 (Fig. 4D) and BCl-6. In situ hybridization revealed cells positive for EBV-encoded small RNAs (Fig. 4E).

These EBV-positive cells were negative for LMP1 and EBNA2. Light chain restriction was not detected by *in-situ* hybridization and monoclonality was not demonstrated. EBV-viral capsid antigen antibody immunoglobulin G and EBV-nuclear antigen (EBNA) antibody were also increased, and EBV PCR yielded positive results. Based on the presence of EBV infection, abnormal proliferation and infiltration of EBV-positive lymphocytes, lack of monoclonality and the occurrence after temozolomide administration, the diagnosis of OIIA-LPD was considered highly likely. Temozolomide therapy was therefore discontinued.

After ending temozolomide therapy, lymphadenopathy, splenomegaly and pancytopenia gradually resolved over the course of a month, and the patient became afebrile and asymptomatic. Platelet count gradually returned to 121 × 10^3^/µL one month after onset of OIIA-LPD. At the same time point, hemoglobin was 9.4 g/dL and white blood cell count was 3400/µL. Achievement of remission was confirmed and the patient was discharged. Temozolomide was kept on hold.

Four months after the onset of OIIA-LPD, the patient experienced intractable epistaxis. Platelet count decreased to 2 × 10^3^/µL and hemoglobin was 6.3 g/dL at this time, necessitating platelet and red blood cell transfusion. White blood cells were also decreased to 1300/µL. Although histopathological evaluations of the tonsils and bone marrow were inconclusive, relapse of OIIA-LPD was suspected clinically and CHOP chemotherapy was introduced. It was not until 3 weeks later that the platelet count returned to 77 × 10^3^/µL, and hemoglobin and white blood cell count returned to 8.7 g/dL and 2900/µL, respectively. Since then, the patient has undergone vigilant follow-up for 14 months on an outpatient basis. Fortunately, the brainstem glioma has remained radiographically stable without requiring resumption of temozolomide treatment and the patient has not experienced any further recurrence of OIIA-LPD.

The patient provided written informed consent for the case to be reported.

## Discussion

In the present case, the patient experienced pancytopenia together with constitutional symptoms during maintenance temozolomide therapy. As cytopenia was grade 4 according to Common Terminology Criteria for Adverse Events (CTCAE) version 5.0, interruption of maintenance therapy was necessary. Cytopenia is not at all uncommon during temozolomide therapy, but cytopenia to this extent is rare. CTCAE Grade 3/4 hematologic toxicity is usually observed in 0–10.2% of patients on temozolomide therapy, [17–19] and TIAA is a fatal pathology that should be considered among the differential diagnoses when cytopenia is encountered [20]. In the present case, however, the patient also demonstrated splenomegaly and generalized lymphadenopathy, which are not characteristic of TIAA. Light chain restriction was not detected by in-situ hybridization and monoclonality was not demonstrated. EBV-viral capsid antigen antibody immunoglobulin G and EBNA were also increased, and EBV PCR showed positive results. Histopathology revealed atypical lymphocytic infiltration with EBV-positive cells. Based on the presence of EBV infection, abnormal proliferation and infiltration of EBV-positive lymphocytes, lack of monoclonality and the occurrence after temozolomide administration, the diagnosis of OIIA-LPD was considered highly likely. Although hematologic malignancies such as leukemia and myelodysplastic syndrome secondary to temozolomide administration have already been described multiple times [10–13], and a small number of cases of non-Hodgkin’s lymphoma following temozolomide administration have also been reported [21], the relationships of those pathologies to EBV were not described. Neither were any of those cases defined as OIIA-LPD. Hence, this case furthers our understanding of adverse events that can be caused by temozolomide.

EBV positivity has been implicated in contributing to the development of OIIA-LPD [6]. EBV infection is presumed to relate to the pathogenesis through DNA methylation, which can interrupt the expression of tumor suppressor genes [22]. Measurement of plasma EBV-DNA is reportedly useful for disease diagnosis and monitoring [23]. Peak EBV loads in serum are significantly higher in patients with LPD compared to those without the disorder [24]. Although LPD may occur in EBV-negative patients and a negative EBV-DNA load does not always exclude the diagnosis of LPD [25], initial evaluation of EBV-DNA load may be worthwhile when LPD is suspected [23, 24]. While histopathological diagnosis including immunohistochemical staining is prerequisite to definitive diagnosis, typical clinical presentations including constitutional symptoms, lymphadenopathy, splenomegaly and pancytopenia can facilitate diagnosis.

Consideration of the reasons why this patient experienced such a rare complication following temozolomide therapy is important. Although EBV is a remarkably prevalent virus that infects > 90% of the global population [26], only a limited number of patients experience tumorigenesis related to EBV infection [24]. Pancytopenia subsequent to temozolomide therapy might have resulted in immunodeficiency and caused EBV reactivation. The vulnerability of the host immune surveillance system may be a factor that affects tumorigenesis. Another factor may be the specific EBV strain, with more than 70 strains now identified [26]. Certain strains have garnered attention for their high potential of tumorigenic potential [27]. Although the exact risk factors for developing LPDs remain unclear, we presume that the host immune system and EBV strain might have influenced the development of OIIA-LPD in the present case.

**Fig. 5:**
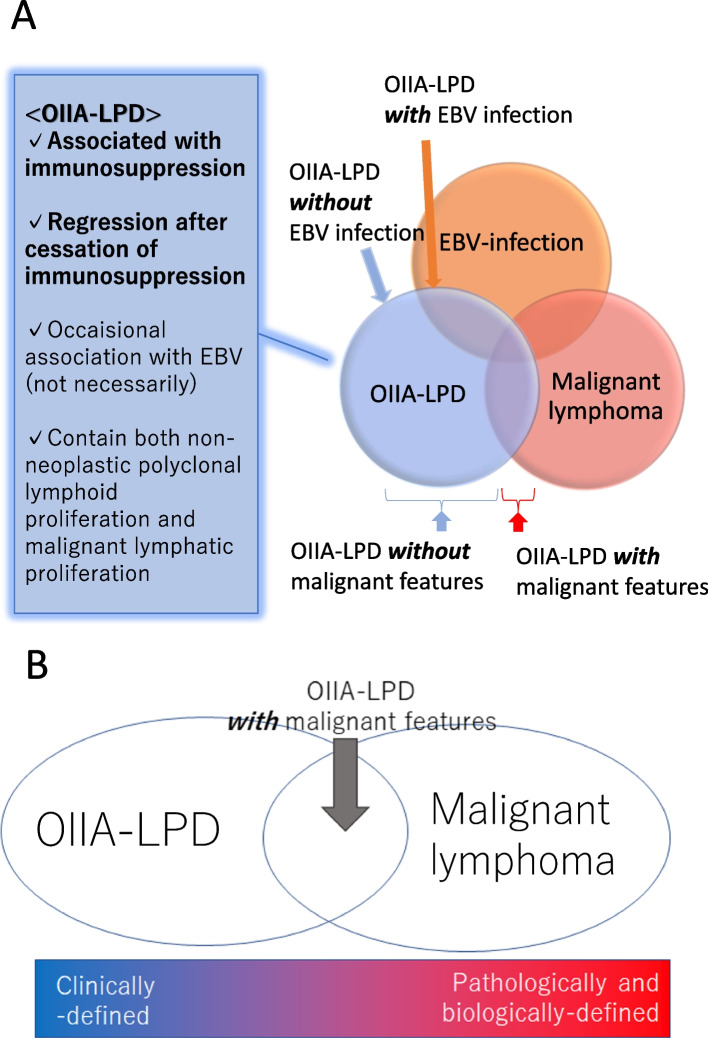
OIIA-LPD is characterized by its association with immunosuppression, and regression after cessation of immunosuppressive agents. OIIA-LPD is occasionally associated with EBV infection, albeit not necessarily. This entity ranges from non-neoplastic lymphoid proliferation to malignant lymphoma (**A**). OIIA-LPD is mainly defined by its clinical pictures, whereas malignant lymphoma is predominantly defined pathologically and biologically (**B**)

In contrast to hematologic malignancies that require intensive chemotherapy for treatment [28], OIIA-LPD has been known to regress either spontaneously or with reduction of the causative agent [4]. Subsequent development of more aggressive OIIA-LPDs, including diffuse large B-cell lymphoma or anaplastic large cell lymphoma, has been observed on rare occasions [2]. The differentiation between OIIA-LPDs and malignant lymphoma may not always be clear in clinical practice; however, the occurrence of the disease during immunosuppression and remission after the discontinuation of temozolomide were characteristic of OIIA-LPDs and not of lymphomas (Fig. 5A). [4] Systemic lymphomas are clearly biologically malignant, while LPD often shows a benign clinical course, such as in this case. OIIA-LPDs are mainly defined by its clinical pictures, whereas malignant lymphomas are predominantly defined by its pathological and biological features (Fig. 5B). Regression of OIIA-LPDs after withdrawal of the causative immunosuppressive drug is one of the unique characteristics of this pathology, so immunosuppressant withdrawal should be attempted as the first step to management [4]. With that said, some cases of OIIA-LPD relapse after temporary remission, or do not even respond to discontinuation of the immunosuppressant [4, 29]. In fact, our case showed a second instance of severe pancytopenia 4 months after withdrawal of temozolomide and required CHOP chemotherapy [30]. The mechanism is explained by the underlying potency of host immunity after discontinuation of the causative drugs; [4] no relapse is observed if the immunity recovers, whereas the significant impairment of immunity may lead to relapse/regrowth events. In fact, our case exhibited profound and prolonged leukocytopenia during and after maintenance temozolomide, which might reflect the impact of immunosuppression caused by induction therapy and maintenance therapy with temozolomide. The efficacy of CHOP chemotherapy against aggressive LPD has been demonstrated previously [4]. Careful monitoring of the clinical and laboratory data and vigilant follow-up are thus the cornerstones to the management of this entity.

Evidence remains scant about when or if temozolomide can be resumed after remission of OIIA-LPD. The guidelines of the Japan College of Rheumatology state that immunosuppressive drugs should be avoided after a patient develops OIIA-LPD [31]. However, temozolomide is the key drug in glioma treatment and has been demonstrated to improve long-term prognosis [7]. Fortunately, the present case showed radiographically stable disease without further administration of temozolomide. Furthermore, MGMT methylation status investigated using DNA from a biopsied specimen of brainstem was low, supporting the decision not to resume treatment. Although our case was fortunate since the patient did not experience relapse of the primary lesion, cases that are intolerant to chemotherapy or experience relapse of the primary lesion would be difficult situations. Re-administration of temozolomide might theoretically lead to aggravation of the OIIA-LPD. Targeted therapy based on gene panel analysis or bevacizumab would be the alternative options. Nevertheless, future studies need to clarify the optimal treatment of glioma itself after the development and regression of OIIA-LPD subsequent to temozolomide administration. In the current situation, the risk of OIIA-LPD relapse needs to be balanced with the risk of glioma progression, paving the way to optimal management for each individual case.

## Conclusions

We have presented a case in which OIIA-LPD developed subsequent to temozolomide therapy. Discontinuation of temozolomide led to rapid regression of the OIIA-LPD. Despite early relapse, the patient remained stable without late relapse in the following 14 months. Although extremely rare during and after temozolomide treatment, severe pancytopenia, splenomegaly and generalized lymphadenopathy raise concerns about OIIA-LPD. Vigilant follow-up should be continued even after remission, since late relapse may occur. Balancing treatments for OIIA-LPD and glioma remains a formidable challenge in this clinical scenario.

## Supplementary Information

**Additional file 1.** 

## Data Availability

All data analyzed during this study are included in this published article.

## Abbreviations

CTCAE: Common Terminology Criteria for Adverse Events
EBV: Epstein-Barr virus
EBNA: Epstein-Barr virus nuclear antigen
HIV: Human immunodeficiency virus
LPD: Lymphoproliferative disorder
MRI: Magnetic resonance imaging
OIIA-LPD: Other iatrogenic immunodeficiency-associated lymphoproliferative disorders
PCR: Polymerase chain reaction
sIL-2R: Soluble interleukin-2 receptor
TIAA: Temozolomide-induced aplastic anemia
WHO: World Health Organization
