# Supplementary figures and images for: Lymphoproliferative disorder during temozolomide therapy; a representative case of a formidable complication and management challenges
Source: BMC Neurol. 2023 Jun 9;23:224. doi: 10.1186/s12883-023-03274-8 (PMC10251676; doi:10.1186/s12883-023-03274-8)

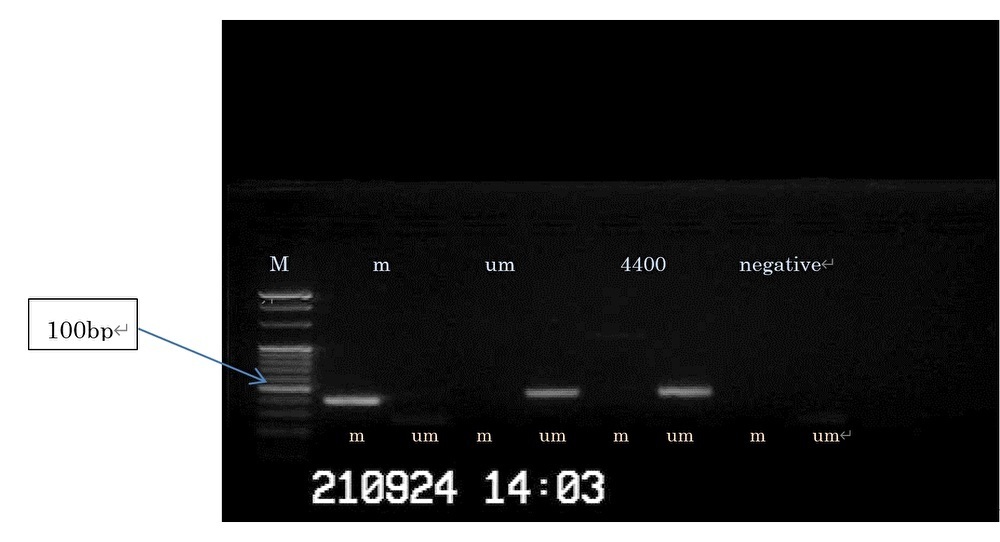

Supplement: Supplementary file 1 — Additional file 1. [file 12883_2023_3274_MOESM1_ESM.jpg]
